# Perceived coercion in psychiatric hospital admission: validation of the French-language version of the MacArthur Admission Experience Survey

**DOI:** 10.1186/s12888-017-1519-4

**Published:** 2017-11-06

**Authors:** Philippe Golay, Imane Semlali, Hélène Beuchat, Valentino Pomini, Benedetta Silva, Laurent Loutrel, Jacques Thonney, Sylfa Fassasi Gallo, Stéphane Morandi, Charles Bonsack

**Affiliations:** 1 Community Psychiatry Service, Department of Psychiatry, Lausanne University Hospital (CHUV), Consultations de Chauderon, Place Chauderon 18, 1003 Lausanne, Switzerland; 2 General Psychiatry Service, Treatment and Early Intervention in Psychosis Program (TIPP–Lausanne), Lausanne University Hospital (CHUV), Lausanne, Switzerland; 3 Institute of Psychology, Faculty of Social and Political Science, University of Lausanne, Lausanne, Switzerland; 4 North Vaud Psychiatric Centre, Department of Psychiatry, Lausanne University Hospital (CHUV), Yverdon, Switzerland

**Keywords:** Perceived coercion, Compulsion, Validation, Item response model, Reliability, Validity

## Abstract

**Background:**

The MacArthur Admission Experience Survey (AES) is a widely used tool to evaluate the level of perceived coercion experienced at psychiatric hospital admission. The French-language AES was prepared using a translation/back-translation procedure. It consists of 16 items and 3 subscores (perceived coercion, negative pressures and voice). This study aimed to assess the psychometric properties of the French-language AES.

**Methods:**

152 inpatients were evaluated. Reliability was estimated using internal consistency coefficients and a test–retest procedure. Internal validity was assessed using a two-parameter logistic item response model. Convergent validity was estimated using correlations between the AES scores and the Coercion Ladder (CL), the Coercion Experience Scale (CES) and the Global Assessment of Functioning (GAF) scale. Discriminatory power was evaluated by comparing the scores of patients undergoing voluntary or compulsory admission.

**Results:**

The French-language AES showed good internal consistency and test–retest reliability. Internal validity of the three-factor model was excellent. Correlations between AES and CL, CES and GAF scores suggested good convergent validity. AES scores were significantly higher among patients subject to compulsory psychiatric hospital admission than among those admitted voluntarily.

**Conclusions:**

Overall, the French-language version of the AES demonstrated very good psychometric proprieties.

## Background

In 1793, Philippe Pinel advocated freeing people with mental disorders from their chains and giving them back their dignity. Despite his desire to make psychiatric treatment more humane, he could not get rid of coerced institutionalisation and, ten years later, he was to recommend the use of the straitjacket as a new form of treatment. Since then, politicians, jurists and mental health professionals have been caught between their duty to respect patients’ fundamental rights and autonomy and the necessity to protect patients (and others) from themselves. Involuntary psychiatric treatments aim to protect people with mental disorders and improve their health status [1]. However, some authors have argued that there is scarce evidence of any patient benefits from compulsory inpatient admission [2]. Indeed, coercive measures may have severe, enduring negative effects on the people targeted, such as increased use of future coercive measures [3, 4], worse quality of life [5], reduced treatment adherence [6] and lower satisfaction with care [7].

A corpus of scientific literature has demonstrated that patients’ feelings of being coerced into psychiatric treatment—their level of perceived coercion—negatively influence their prognoses, more than the formal coercive measures themselves [8]. Perceived coercion is not exclusively related to formal coercive measures or the patient’s legal status at admission [9]; it also depends on the amount of information shared with the patient, participation in medical decision-making and a lack of knowledge about legal issues. Moreover, informal coercion, such as leverage, can influence the level of perceived coercion [10]. Even voluntary patients can therefore report high levels of perceived coercion [11, 12]. Patients’ perceptions of coercion can be influenced by their gender, the severity of symptoms, their level of social functioning and the quality of their therapeutic relationships [13, 14]. Perceived coercion damages the patient’s perception of the therapeutic relationship [15], and lower levels of perceived coercion are linked to higher treatment satisfaction [16]. The long-term impact of high levels of perceived coercion on patients is nevertheless controversial and needs further evaluation [17].

Several approaches have been developed to decrease levels of perceived coercion. These have highlighted that patient empowerment, cooperation with professionals [18], a moral approach to coercion [19], and respect for patients’ freedom of choice and values [20] are determining factors.

One of the most widely used tools for studying perceived coercion is the Admission Experience Survey (AES) short form developed for the MacArthur Coercion Study [21]. More precisely, this 16-item dichotomous (true-or-false) questionnaire was derived from a structured interview (the *MacArthur Admission Experience Interview*) so that patients’ perceptions of psychiatric hospital admission could be obtained rapidly using a paper and pencil. Among other scoring variants, three subscales were proposed: the *Perceived Coercion* score focuses on freedom, choice, initiative, control and influence over coming into hospital; the *Negative Pressures* score focuses on being forced, threatened or physically forced to come into hospital; and the *Voice* score focuses on having a chance to voice an opinion about coming into hospital [21–24]. One item (#9) was eventually dropped from these subscales, and the last item (#16) is a series of adjectives used to evaluate the patient’s affective reaction to hospitalisation. Items and scoring instructions from the original English version are available on the MacArthur Research Network on Mental Health and the Law website [22].

To the best of our knowledge, there is no specific French-language tool available for the study of perceived coercion. This current lack of proper research tools makes any investigation of coercion in French-speaking countries difficult at best. This study’s goal, therefore, was to assess the psychometric properties of a French-language version of the AES. Validation of a French-language perceived coercion scale will promote further research projects on this topic in French-speaking countries and enable an international comparison of results. A better understanding of the factors influencing patients’ perceptions of coercion and, consequently, its impact on their welfare and prognoses, will help us to develop new, alternative models of care that enable a reduction in the use of compulsory admission.

## Methods

### Participants

A total of 152 patients were recruited during their hospitalisation in Lausanne University Hospital’s Department of Psychiatry. Mean age was 41.7 (SD = 12.7) years old, and 52.6% (80) of participants were women. The average level of general functioning, as assessed using the Global Assessment of Functioning (GAF) scale, was 41.8 (SD = 12.9), and 30.3% (46) of patients were admitted involuntarily, according to their caregivers. Thus, 106 patients were admitted on a voluntary basis. The majority of patients (69.1%; 105) were born in Switzerland. Primary diagnoses based on the International Statistical Classification of Diseases and Related Health Problems 10th Revision (ICD-10) were: 36.2% (55) schizophrenia, 31.6% (48) depression, 11.2% (17) personality disorder, 6.6% (10) mania, 5.3% (8) anxiety and stress-related disorders, 5.3% (8) drug use and 3.9% (6) alcohol use.

### Measures

#### French-language version of the MacArthur admission experience survey short form

The AES was translated into French by CB, SM and PG and back-translated into English by an independent professional translator. One of the scale’s original authors (SKH) checked the back-translation against the English version. Although not 100% identical, all the item translations were considered very accurate and similar in meaning. The original author therefore endorsed the French-language version without further modifications (Table 1).

**Table 1:** French-language version of the AES

Instructions	*Répondez s’il vous plaît soit “VRAI” ou “FAUX” à chaque énoncé. Essayez de répondre à chaque question individuellement, sans tenir compte des ressemblances avec d’autres questions.*
Items	
*1*	*Je me suis senti(e) libre de faire ce que je voulais en venant à l’hôpital.*
2	*On a essayé de me forcer à venir à l’hôpital*
3	*J’ai eu suffisamment l’occasion de dire si j’étais d’accord de venir à l’hôpital*
4	*J’ai choisi de venir à l’hôpital*
5	*J’ai pu dire ce que je voulais à propos de venir à l’hôpital*
6	*Quelqu’un m’a menacé pour me faire venir à l’hôpital*
7	*C’était mon idée de venir à l’hôpital*
8	*Quelqu’un a essayé de m’obliger physiquement à venir à l’hôpital*
9	*Personne ne semblait vouloir savoir si j’étais d’accord de venir à l’hôpital*
10	*J’ai été menacé d’être hospitalisé contre mon gré*
11	*On m’a dit qu’on m’obligerait à venir à l’hôpital*
12	*Personne n’a essayé de me forcer à venir à l’hôpital*
13	*Mon opinion quant au fait de venir à l’hôpital n’a eu aucune importance*
14	*J’ai eu beaucoup de contrôle sur le fait de venir ou non à l’hôpital*
15	*J’ai eu plus d’influence que quiconque sur la décision de venir ou non à l’hôpital*
16	*Comment avez-vous ressenti le fait d’être admis(e) à l’hôpital*? *a. En colère.* *b. Triste.* *c. Content(e).* *d. Soulagé(e).* *e. Troublé(e).* *f. Effrayé(e).*

Note. *Items and scoring instructions from the original English version are available on the MacArthur Research Network on Mental Health and the Law website http://www.macarthur.virginia.edu/shortform.html;* [22]

#### Coercion ladder

The Coercion Ladder [25] was originally adapted from the Cantril Ladder [26]. It is a visual analogue tool on which the patient is asked to mark the degree of perceived coercion on a scale of 1 (Minimum use of coercion*—I came totally on my own will and initiative*) to 10 (Maximum use of coercion). The Coercion Ladder’s test–retest reliability in this study was good (*r* = 0.77; ICC [1, 2] = 0.77).

#### Coercion experience scale (CES)

The CES [27] is a 35-item scale designed to measure patients’ experiences of coercive measures. The first two items are 0–100 visual analogue scales designed to evaluate the extent to which patients remember coercive measures (item 1) and the extent to which these were considered stressful (item 2). All other items are five-point Likert-type scales. We selected the *Coercion* subscore and the second item (stress) score as indicators of coercion. We also computed a total score in order to represent the overall experience of coercion. The French-language version of the CES was back-translated by a professional translator and was approved by the original authors. The test–retest reliability of the CES scores used in the present study ranged from acceptable (item 2 – *r* = .62; ICC [1, 2] = 0.61) to good (Coercion subscore – *r* = 0.80; ICC [1, 2] = 0.80; total score – r = 0.80, ICC [1, 2] = 0.81).

#### Global assessment of functioning (GAF)

The GAF [28] is a numerical scale taken from the Diagnostic and Statistical Manual of Mental Disorders Fourth Edition (DSM-IV) and designed to evaluate an individual’s social, occupational and psychological functioning. It ranges from 1 (severely impaired) to 100 (extremely high functioning).

### Procedure

The reliability of the French-language AES scores was assessed using a test–retest approach with an interval of between 2 and 14 days; 43 patients participated in the retest. Internal consistency estimates were also computed on the basis of the first assessment. To assess the internal validity of the French-language AES scores, we tested the original three-factor AES scoring model by loading items 1, 4, 7, 14 and 15 on the *Perceived Coercion* factor, items 2, 6, 8, 10, 11 and 12 on the *Negative Pressures* factor and items 3, 5 and 13 on the *Voice factor*. As with the original scale, item #9 was discarded. Because the three factors were highly correlated, a single-factor model including an overall perceived coercion factor was also estimated using all 14 items. In this total score, the items related to *Voice* were reversed because they indicated less coercion. To estimate convergent validity, several indicators were used to study the relationship between AES scores and other scales. We hypothesised that the AES *Perceived Coercion* score, *Negative Pressures* score and *Total* score would be positively correlated with the Coercion Ladder score, the *CES’s* second item (stress measured on a 0–100 scale), the CES *Coercion* subscore and the CES *Total* score. We hypothesised that these scores would also be negatively correlated with the *GAF* score under the hypothesis that higher-functioning individuals may experience less coercion from mental health professionals. Finally, we hypothesised that the AES *Voice* score would be negatively correlated with the CES, CL and GAF scores (i.e. more voice was associated with less coercion or better functioning).

### Statistical analysis

#### Reliability

The reliability of the AES subscales was estimated using McDonald’s model-based Omega (ω) [29] and Cronbach’s alpha (α) coefficients. The test–retest reliability was estimated using both Pearson and intraclass correlation coefficients using a two-way random-effects model and the absolute agreement definition (ICC [1, 2]). Reliability coefficients above .70 were considered satisfactory; above .80 were considered good; and above .90 were considered excellent [29, 30]. The presence of systematic changes between first and second assessments was evaluated using paired-sample Student t-tests.

#### Internal validity

Due to the items’ dichotomous nature, internal validity was estimated using two-parameter logistic (2PL) item response models. All models were estimated using a robust weighted least squares estimator with adjustments for the mean and variance (WLSMV). First, a three-factor model was estimated, as was a single-factor model including a general perceived coercion factor. These two models were compared with a robust chi-square test using the DIFFTEST procedure. Several indicators of model fit were used: the root mean square error of approximation (RMSEA), the comparison fit index (CFI) and the Tucker–Lewis fit index (TLI). RMSEA values ≤0.06, and CFI and TLI values ≥0.95, were interpreted as good fits, whereas RMSEA values ≤0.08, and CFI and TLI values ≥0.90 were considered as indicating acceptable fit [31].

#### Convergent validity

The convergent validity coefficients between the French-language AES scores and the other scales were estimated using Pearson correlation coefficients. There are no well-established criteria for the interpretation of convergent validity coefficients. Given that the upper bound of any validity coefficient is the square root of the score reliability, the acceptable range is usually lower than for reliability coefficients. In the present study, we interpreted correlation coefficients between .40 and .60 as good and any values higher than .30 (a medium effect size, according to Cohen [32]) as satisfactory.

#### Discrimination

To test whether the French-language AES could discriminate between voluntarily and involuntarily admitted patients, their average scores were compared using an independent sample Student t-test. Our hypothesis was that the latter group would report higher levels of coercion. All statistical tests were two-tailed, and a significance level was set at α = 0.05. All statistical analyses were performed using the Mplus statistical package (version 7.4) and IBM SPSS 23.

## Results

### Reliability

Internal consistency and test–retest reliability estimates (Table 2) were satisfactory to excellent [29, 30]. Comparisons between scores from the first and second assessments revealed no significant changes.

**Table 2:** Reliability of AES scores

	Internal Consistency (*N* = 152)		Test–retest reliability (*N* = 43)	
	McDonald’s ω	Cronbach’s α	Pearson’s r	ICC (2,1)
*Perceived Coercion subscale*	.927	.796	.764**	.766**
*Negative Pressures subscale*	.947	.837	.772**	.774**
*Voice Subscale*	.919	.787	.780**	.782**
*Total Scale*	.974	.913	.889**	.890**

Note. **p < .01

### Internal validity

**Fig. 1:**
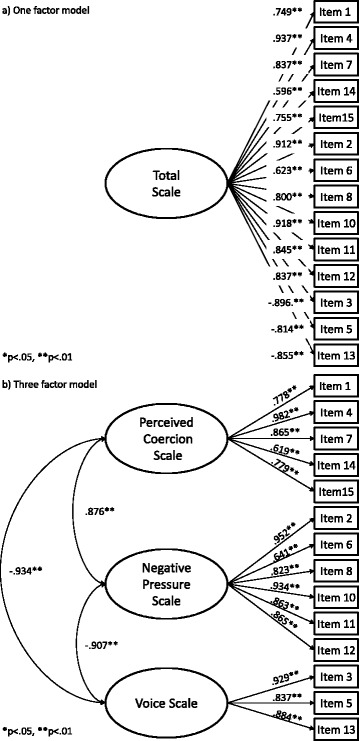
**a** One- and **b** three-factor models of the AES scale

The three-factor model’s fit was excellent (Table 3). All factor loadings were statistically significant, and the three factors were highly correlated (76%–87% of shared variance) with each other (Fig. 1).

**Table 3:** Comparisons of model fit and IRT parameterisation for the AES

Model	χ^2^	df	*p*-value	RMSEA	90% C.I. for RMSEA	CFI	TLI
One-factor model	112.326	77	.005	0.055	0.031–0.076	0.987	0.985
Three-factor model	94.235	74	.056	0.042	0.000–0.066	0.993	0.991
IRT parameterisation		One-factor model			Three-factor model		
		Item difficulty	Item discrimination		Item difficulty	Item discrimination	
Item 1		−0.347	1.130		−0.334	1.238	
Item 2		0.374	2.223		0.358	3.110	
Item 3		0.613	−2.018		−0.591	2.510	
Item 4		0.241	2.682		0.230	5.199	
Item 5		0.733	−1.401		−0.713	1.530	
Item 6		1.681	0.796		1.633	0.835	
Item 7		−0.100	1.530		−0.097	1.724	
Item 8		1.023	1.333		0.994	1.449	
Item 10		0.503	2.315		0.495	2.614	
Item 11		0.538	1.580		0.527	1.708	
Item 12		0.070	1.530		0.068	1.724	
Item 13		0.312	−1.649		−0.302	1.891	
Item 14		−0.059	0.742		−0.057	0.788	
Item 15		−0.180	1.151		−0.175	1.242	

Note. *IRT* Item Response Theory, *df* degree of freedom, *RMSEA* Root Mean Square Error of Approximation, *C.I.* Confidence Interval, *CFI* Comparative Fit Index, *TLI* Tucker–Lewis Index

The more restrictive one-factor model also showed very good fit. Again, all factor loadings were supported (Fig. 1). Direct comparison between the two models indicated that the three-factor solution was preferable (Δχ2 = 16.986; Δdf = 3; *p* < .001).

**Fig. 2:**
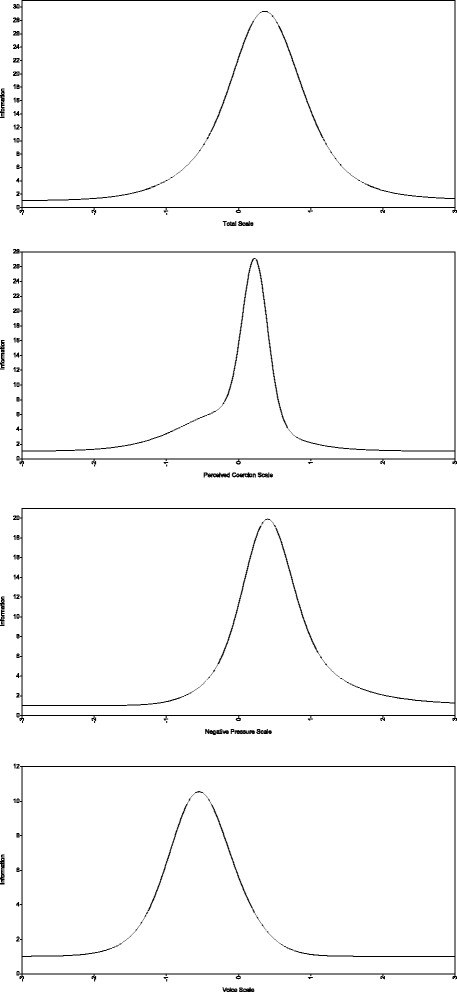
Total Information Curves for the one- and three-factor models of the AES scale

For reference, and as a complement to the factor loadings, Table 3 gives each item’s discrimination and difficulty, for both models, as an Item Response Theory metric. The Total Information Curves (Fig. 2) indicate the amount of information in the latent scores that were explained by the subscale items across different levels of the latent construct.

### Convergent validity

All correlation coefficients were significant and in the expected direction (Table 4). As indicated by their substantive correlations, patients who reported a higher level of coercion on the CES and the CL also tended to report higher scores on the AES. GAF scores were also moderately correlated with AES scores.

**Table 4:** Convergent validity of the AES scores

	Coercion Ladder	CES			GAF
		Item 2 (stress)	Coercion score	Total score	
*Perceived Coercion subscale*	.560**	.492**	.578**	.463**	−.379**
*Negative Pressures subscale*	.687**	.609**	.631**	.583**	−.196*
*Voice subscale*	−.653**	−.560**	−.568**	−.442**	.217**
*Total Scale*	.706**	.622**	.679**	.592**	−.304**

Note. * = p < .05, ** = p < .01

### Discrimination according to admission status

The differences between patients admitted voluntarily and involuntarily were large for all four AES scores. Involuntarily admitted patients scored significantly higher on the *Perceived Coercion* scale (t(148) = 6.748; *p* < .001; d = 1.26), the *Negative Pressures* scale (t(149) = 6.740; p < .001; d = 1.18) and the *Total* scale (t(147) = 7.973; p < .001; d = 1.45). They scored lower on the *Voice* scale (t(148) = −6.198; p < .001; d = −1.08).

## Discussion

The reliability of the French-language Admission Experience Survey (AES) scores was very satisfactory. Furthermore, these scores underwent no systematic changes at the second assessment, which is a desirable feature in evaluation settings. The internal validity of the one- and three-factor AES models was shown to be very good. Correlations between the three factors suggested that *Perceived Coercion*, *Negative Pressures* and *Voice* shared a lot of variance. This was in line with other studies that have shown that perceived coercion was also associated with the use of negative pressures such as threats and physical force. Additionally, perceived coercion was found to be inversely related to a patient’s sense of procedural justice, that is, the patient’s belief that he had been able to voice his opinion and had been treated with fairness, concern and respect [9]. Although the computation of a single total coercion score was perfectly adequate, the comparison between the one- and three-factor models suggested that *Perceived Coercion*, *Negative Pressures* and *Voice* should not be considered as indistinguishable. Despite the large amount of shared variance, there are further benefits to conceptualising an AES with three subscales. The high correlation between subscales only suggests that patients are likely to have similar scores on average. However, this will not always be the case, and differences may highlight important clinical situations. Furthermore, examination of the Total Information Curves suggested that the AES *Negative Pressures* subscore provided more information on respondents who scored above average on the latent trait, whereas the AES *Voice* score was more informative about respondents with below-average latent scores. Consequently, using the subscales may add to the AES’s sensitivity with patients presenting different levels of perceived coercion.

Correlations between the French-language AES scores and the Coercion Ladder score, the Coercion Experience Scale and the Global Assessment of Functioning scale were largely in line with expectations, suggesting that the French-language version of the AES is a valid measure of perceived coercion. The correlation patterns between each of the four AES scores and the other scales were very similar, which is not surprising given the high correlation between the factors. However, the three-factor model’s superiority over the single-factor model suggests that distinguishing between these three subscores could be useful, adding information above and beyond that provided by a single summary score.

Finally, all four scores derived from the French-language AES were able to discriminate between patients who had been voluntarily and involuntarily admitted to hospital, which confirmed our hypothesis.

## Conclusions

The French-language version of the AES demonstrated very good psychometric properties. The validation of this questionnaire was a mandatory first step towards testing other hypotheses and conducting future clinical or interventional studies. We hope that it will promote the development of further research projects on these topics in French-speaking countries and comparisons with the English or German-speaking countries where these tools are already in use.

## Abbreviations

2PL: Two-parameter logistic
AES: MacArthur Admission Experience Survey
CES: Coercion Experience Scale
CFI: Comparison fit index
CL: Coercion Ladder
DSM-IV: Diagnostic and Statistical Manual of Mental Disorders Fourth Edition
GAF: Global Assessment of Functioning
ICC: Intraclass Correlation Voefficient
ICD-10: International Statistical Classification of Diseases and Related Health Problems 10th Revision
RMSEA: Root Mean Square Error of Approximation
TLI: Tucker–Lewis fit index
WLSMV: Robust Weighted Least Squares Estimator with Adjustments for the Mean and Variance
